# Supplementary material for: Candidate genes under selection in song sparrows co-vary with climate and body mass in support of Bergmann’s Rule
Source: Nat Commun. 2023 Nov 7;14:6974. doi: 10.1038/s41467-023-42786-2 (PMC10630373; doi:10.1038/s41467-023-42786-2)
Supplement: Supplementary file 1 — Supplementary Information [file 41467_2023_42786_MOESM1_ESM.pdf]

CANDIDATE GENES UNDER SELECTION IN SONG SPARROWS CO-VARY WITH CLIMATE AND BODY MASS IN SUPPORT OF BERGMANN'S RULE

Katherine Carbeck, Peter Arcese, Irby Lovette, Christin Pruett, Kevin Winker, and Jennifer Walsh

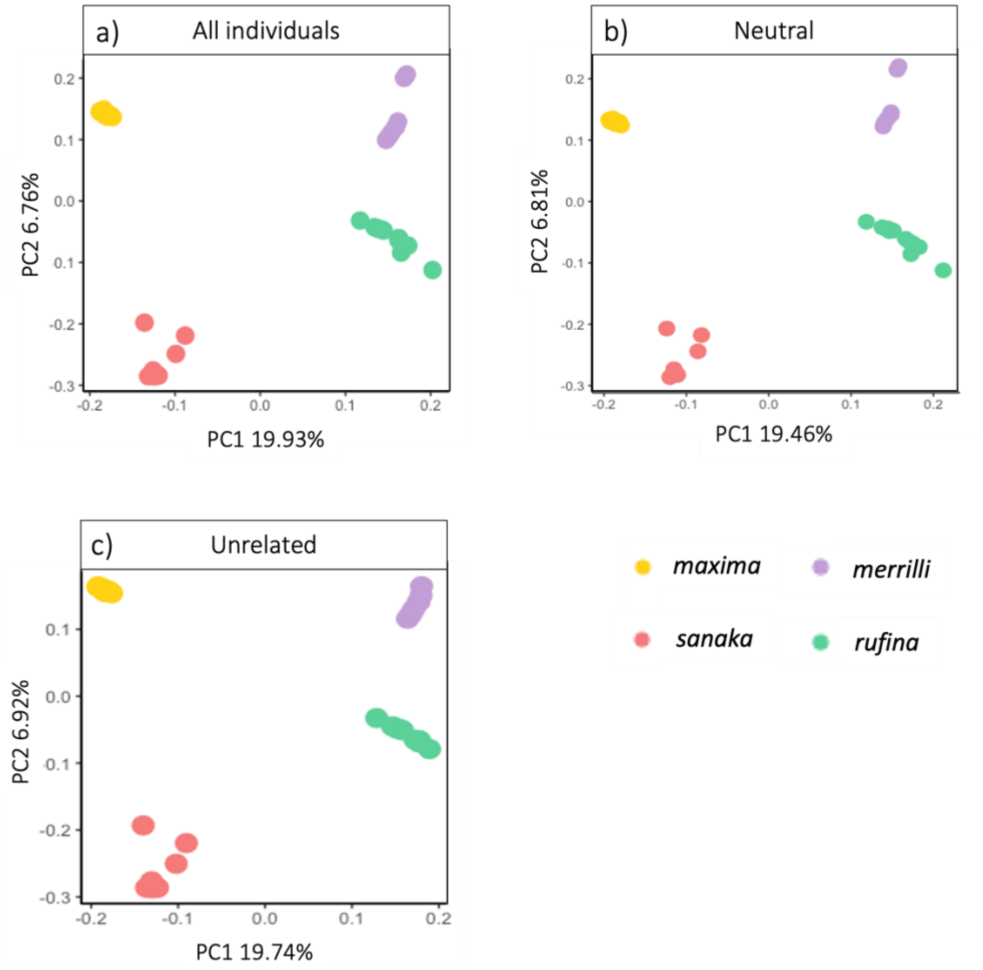

Supplementary Figure 1. Genome-wide patterns of divergence between large- and small-bodied subspecies of song sparrows for: a) all individuals, b) putatively neutral genomic regions (i.e., excluding the coding sequence region and 100,000 bp on each side), and c) unrelated individuals only (excluding three related individuals).

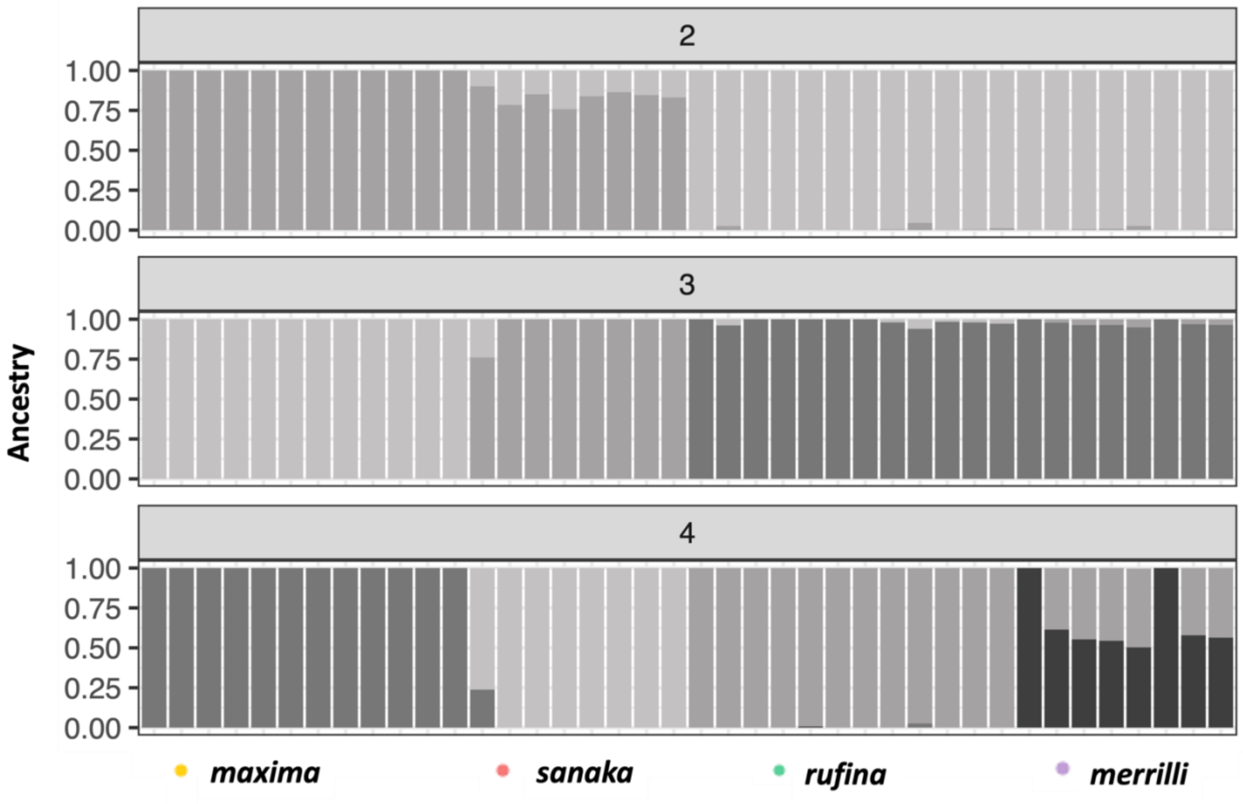

Supplementary Figure 2. Admixture plots based on 1,989,848 SNPs show subspecies' genomic groupings among song sparrows in Alaska and British Columbia at population settings of *K* = 2 to *K* = 4. While the most probable grouping occurs at K = 2 (see text), signals of genomic clustering up to K = 4 (cross validation rates for K=2-4: 0.579; 0.605; 0.638, respectively).

Supplementary Table 1. Pairwise F_{ST} estimates of northern subspecies of *Melospiza melodia* based on 13,089,663 SNPs (whole-genome data). Estimates were generated in VCFtools.

	maxima	*sanaka*	*rufina*	*merrilli*
maxima	0			
sanaka	0.13587	0		
rufina	0.20494	0.17585	0	
merrilli	0.24673	0.15942	0.03477	0

Supplementary Table 2. List of outlier regions (F_{ST} estimates greater than 99.9th percentile above the mean) that appear in all, three, and two large- and small-bodied subspecies comparisons (grey fill) with *maxima-sanaka* and *merrilli-rufina* pairwise F_{ST} included for a control comparison (white fill). Table includes scaffold ID and position for outlier SNPs, chromosome aligned to the zebra finch (*Taeniopygia guttata*) reference genome, the comparisons in which the region was elevated, associated F_{ST} estimate, the candidate gene associated with the SNP, *E*-value from the BLAST results, and putative biological function of the identified candidate gene.

Scaffold	Chr	Start position	Comparison	FST	Candidate gene	E value	GO Biological Function
Contig391_pilon	17	36,121	*sanaka-merrilli*	0.857278	FBXW2	0	cellular protein modification process; post-translational protein modification; protein polyubiquitination; proteolysis
			sanaka-rufina	0.744801			
			maxima-rufina	0.75201			
			maxima-merrilli	0.83723			
			maxima-sanaka	0.00537737			
			merrilli-rufina	0.0295779			
Contig391_pilon	17	2,006	*sanaka-merrilli*	0.857278	GARNL3	2.56E-82	regulation of small GTPase mediated signal transduction; activation of GTPase activity
			sanaka-rufina	0.744801			
			maxima-rufina	0.75201			
			maxima-merrilli	0.83723			
			maxima-sanaka	0.00537737			
			merrilli-rufina	0.0295779			
Contig391_pilon	17	57,932	*sanaka-merrilli*	0.857278	RALGPS1	3.12E-63	small GTPase mediated signal transduction; regulation of Ral protein signal transduction; intracellular transduction
			sanaka-rufina	0.744801			
			maxima-rufina	0.75201			
			maxima-merrilli	0.83723			
			maxima-sanaka	0.00537737			
			merrilli-rufina	0.0295779			
Contig391_pilon	17	137,817	*sanaka-merrilli*	0.834524	ZBTB34	0	regulation of transcription by RNA polymerase II
			sanaka-rufina	0.677277			
			maxima-rufina	0.701398			
			maxima-merrilli	0.831221			
			maxima-sanaka	0.00673037			
			merrilli-rufina	0.0655835			
Contig391_pilon	17	103,909	*sanaka-merrilli*	0.817419	ANGPTL2	0	angiogenesis; multicellular organism development
			sanaka-rufina	0.741907			
			maxima-rufina	0.721944			
			maxima-merrilli	0.787453			
			maxima-sanaka	0.0157578			
			merrilli-rufina	0.0195055			
Contig391_pilon	17	154,340	*sanaka-merrilli*	0.790709	ZBTB43	0	regulation of transcription by RNA polymerase II
			sanaka-rufina	0.624765			
			maxima-rufina	0.65555			
			maxima-merrilli	0.787982			
			maxima-sanaka	0.0341744			
			merrilli-rufina	0.0577231			
Contig3361_pilon	2	195,967	*sanaka-merrilli*	0.665418	COL15A1	4.21E-50	angiogenesis; cell adhesion; collagen catabiolic process; collagen fibril organization; endothelial cell morphogensis; extracellular matrix organization; signal transduction
			sanaka-rufina	0.541066			
			maxima-rufina	0.569297			
			maxima-merrilli	0.677299			
			maxima-sanaka	0.0146049			
			merrilli-rufina	0.0189091			
Contig3361_pilon	2	313,205	*sanaka-merrilli*	0.63508	TGFBR1	4.94E-76	apoptotic process; cell differentiation; growth regulation; activation of MAPKK activity; cellular response to growth factor stimulus; skeletal system development and morphogenesis; thymus development
			sanaka-rufina	0.62381			
			maxima-rufina	0.543972			
			maxima-merrilli	0.688921			
			maxima-sanaka	0.0628615			
			merrilli-rufina	0.0289775			
Contig1534_pilon	3	27,235,878	*sanaka-merrilli*	0.606752	TAF1A	1.69E-60	positive regulation of gene expression, epigenetic; termination of RNA polymerase I transcription; transcription by RNA polymerase I; transcription by RNA polymerase II; transcription initiation from RNA polymerase I promoter
			sanaka-rufina	0.614808			
			maxima-rufina	0.620312			
			maxima-merrilli	0.611122			
			maxima-sanaka	0.00088553			
			merrilli-rufina	0.00393105			
Contig3361_pilon	2	118,419	*sanaka-merrilli*	0.668557	TRIP13	2.98E-69	double-strand break repair; female meiosis I; male meiosis I; meiotic recombination checkpoint; mitotic spindle assembly checkpoint; oocyte maturation; oogenesis; reciprocal meiotic recombination; spermatid development; spermatogenesis; synaptonemal complex assembly; transcription by RNA polymerase II
			sanaka-rufina	0.506722			
			maxima-merrilli	0.656435			
			maxima-sanaka	0.0539854			
			merrilli-rufina	0.0614327			
Contig3361_pilon	2	116,714	*sanaka-merrilli*	0.668557	BRD9	3.35E-99	chromatin organization; regulation of transcription by RNA polymerase II
			sanaka-rufina	0.506722			
			maxima-merrilli	0.656435			
			maxima-sanaka	0.0539854			
			merrilli-rufina	0.0614327			
Contig1534_pilon	3	27,295,063	*maxima-merrilli*	0.574309	AIDA	3.13E-47	determination of ventral identity; dorsal/ventral pattern formation; negative regulation of determination of dorsal identity, JNK cascade, JUN kinase activity, and protein-containing complex assembly.
			maxima-rufina	0.614685			
			maxima-sanaka	0.132394			
			merrilli-rufina	0.00594764			
Contig1534_pilon	3	27,250,001	*maxima-merrilli*	0.574309	Unknown gene ENSTGUP00000002762		
			maxima-rufina	0.614685			
			maxima-sanaka	0.132394			
			merrilli-rufina	0.00594764			
Contig277_pilon	2	150,001	*sanaka-merrilli*	0.693213	TPPP	2.10E-85	astral microtubule organization, microtubule bundle formation, microtubule nucleation by microtubule organizing center, myelin assembly, negative regulation of tubulin deacetylation, oligodendrocyte development, positive regulation of myelination, positive regulation of protein polymerization, regulation of microtubule cytoskeleton
			maxima-merrilli	0.648498			
			maxima-sanaka	0.0497578			
			merrilli-rufina	0.111646			
Contig76_pilon	1	25,778,468	*maxima-merrilli*	0.561215	USP16	5.74E-53	cell cycle; cell division; cellular response to DNA damage stimulus; transcription; Ubl conjugation pathway
			maxima-rufina	0.678335			
			maxima-sanaka	0.283981			
			merrilli-rufina	0.0251675			
Contig76_pilon	1	25,736,638	*maxima-merrilli*	0.636203	LTN1	1.12E-84	protein autoubiquitination, rescue of stalled ribosome, ribosome-associated ubiquitin-dependent protein catabolic process
			maxima-rufina	0.680352			
			maxima-sanaka	0.268182			
			merrilli-rufina	0.08308			
Contig76_pilon	1	25,635,649	*maxima-merrilli*	0.628244	C3ORF52	9.91E-47	endoplasmic reticulum membrane; integral component of membrane
			maxima-rufina	0.526564			
			maxima-sanaka	0.225669			
			merrilli-rufina	0.107123			

Supplementary Table 3. Table summarizing the linear regression analysis for each SNP and allele frequency and body mass. The table includes the SNP for which the linear regression was performed, the intercept and slope with 95% Confidence Intervals, the coefficient of determination (R-Squared), degrees of freedom (df), F-statistic, p-value, Effect Size, Max Hat Value which represents the maximum leverage value across all observations, and Max DF Beta Values which measures how much the intercept and slope coefficient changes when an observation is removed.

SNP	Intercept	Intercept Lower 95%	Intercept Upper 95%	Slope	Slope Lower 95%	Slope Upper 95%	R-Squared	df	F-statistic	p-value	Effect Size	Max Hat Value	Max DFBeta Intercept	Max DFBeta Slope
Contig391_pilon_122228	20.64	18.26	22.90	28.55	18.26	22.90	0.953	7	142.66	0.0000066	3.31	0.579	0.928	0.238
Contig391_pilon_19152	21.23	18.02	20.45	28.36	18.02	20.45	0.911	7	71.85	0.0000629	3.31	0.556	1.85	0.189
Contig391_pilon_86656	21.23	18.01	19.22	26.65	18.01	19.22	0.911	7	71.85	0.0000629	3.31	0.546	1.854	0.189
Contig3361_pilon_255126	21.27	18.00	24.81	34.69	18.00	24.81	0.908	7	68.93	0.0000719	3.32	0.606	1.674	0.333
Contig3361_pilon_255218	21.34	17.91	25.06	35.82	17.91	25.06	0.898	7	61.92	0.0001013	3.32	0.656	1.377	0.566
Contig3361_pilon_262569	20.95	17.46	22.54	32.26	17.46	22.54	0.898	7	61.62	0.0001029	3.31	0.732	0.855	0.92
Contig391_pilon_61835	21.56	17.28	19.24	31.44	17.28	19.24	0.841	7	37.11	0.0004951	3.31	0.696	0.805	1.187
Contig391_pilon_57980	21.68	17.14	19.86	34.15	17.14	19.86	0.82	7	31.96	0.0007721	3.32	0.729	0.71	1.226

Supplementary Table 4. Table summarizing the linear regression analysis for each SNP and allele frequency and average winter and summer temperature. The table includes the SNP for which the linear regression was performed, the intercept and slope with 95% Confidence Intervals, the coefficient of determination (R-Squared), degrees of freedom (df), F-statistic, p-value, Effect Size, Max Hat Value which represents the maximum leverage value across all observations, and Max DF Beta Values which measures how much the intercept and slope coefficient changes when an observation is removed.

SNP	Intercept	Intercept Lower 95%	Intercept Upper 95%	Slope	Slope Lower 95%	Slope Upper 95%	R-Squared	df	F-statistic	p-value	Effect Size	Max Hat Value	Max DFBeta Intercept	Max DFBeta Slope
Contig391_pilon_122228	13.49	11.35	-14.22	-9.142	11.35	-14.22	0.722	7	18.15	0.00375	3.84	0.579	0.536	0.643
Contig391_pilon_19152	13.26	10.92	-14.59	-8.853	10.92	-14.59	0.655	7	13.32	0.00819	3.85	0.556	0.507	0.536
Contig391_pilon_86656	13.26	10.93	-13.71	-8.326	10.93	-13.71	0.657	7	13.38	0.00809	3.84	0.546	0.508	0.539
Contig3361_pilon_255126	13.24	10.88	-17.89	-10.785	10.88	-17.89	0.648	7	12.87	0.00888	3.87	0.606	0.504	0.516
Contig3361_pilon_255218	13.21	10.82	-18.59	-11.089	10.82	-18.59	0.636	7	12.22	0.01006	3.87	0.656	0.498	0.812
Contig3361_pilon_262569	13.37	11.03	-16.70	-10.208	11.03	-16.70	0.664	7	13.82	0.00748	3.86	0.732	0.5	2.031
Contig391_pilon_61835	13.19	10.79	-16.87	-10.02	10.79	-16.87	0.631	7	11.97	0.01055	3.86	0.696	0.497	0.981
Contig391_pilon_57980	13.15	10.72	-18.57	-10.917	10.72	-18.57	0.619	7	11.38	0.01187	3.87	0.729	0.493	1.274

Supplementary Table 5. The top diagonal depicts the mean pairwise F_{ST} for the 8 focal SNPs of northern subspecies of *Melospiza melodia*. The bottom diagonal depicts the number (percentage) of fixed or nearly fixed SNPs (> 0.95 F_{ST}) contained within the 467 SNPs within the 9 candidate genes.

	maxima	*sanaka*	*rufina*	*merrilli*
maxima		0.081	0.763	0.751
sanaka	0 (0%)		0.726	0.713
rufina	14 (3.0%)	26 (5.6%)		0.003
merrilli	158 (33.8%)	115 (24.6%)	0 (0%)	

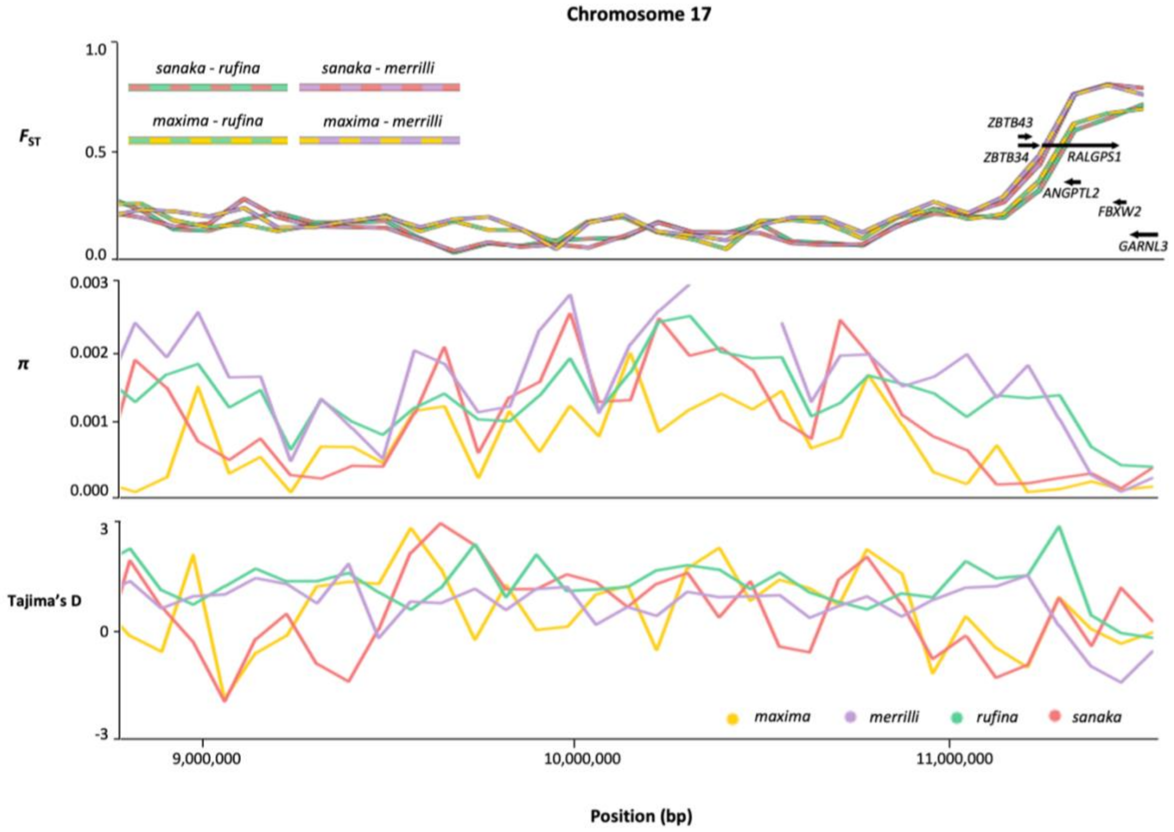

Supplementary Figure 3. Distribution of F_{ST}, π, Tajima's *D* in 25-kb windows, and genes within the divergent windows on Chromosome 17 (contig 391).

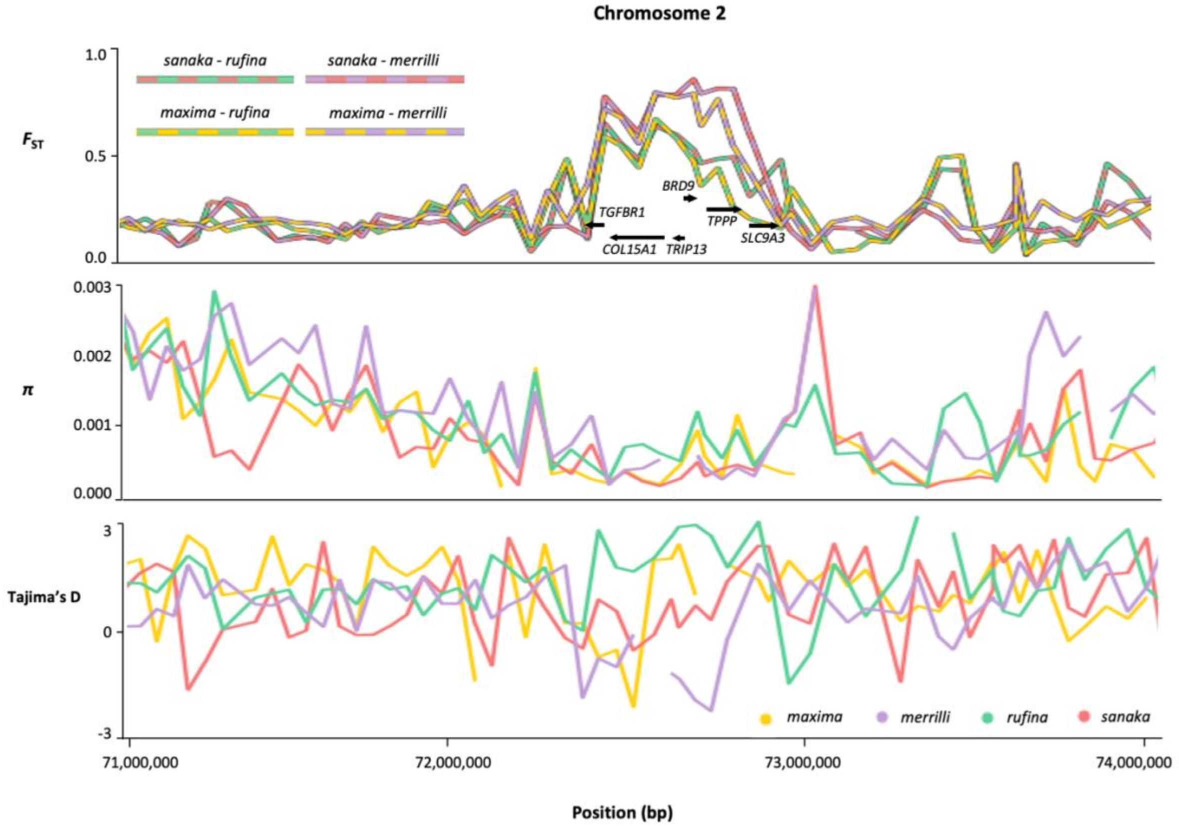

Supplementary Figure 4. Distribution of F_{ST}, π, Tajima's *D* in 25-kb windows, and genes within the divergent windows on Chromosome 2 (contig 3361).

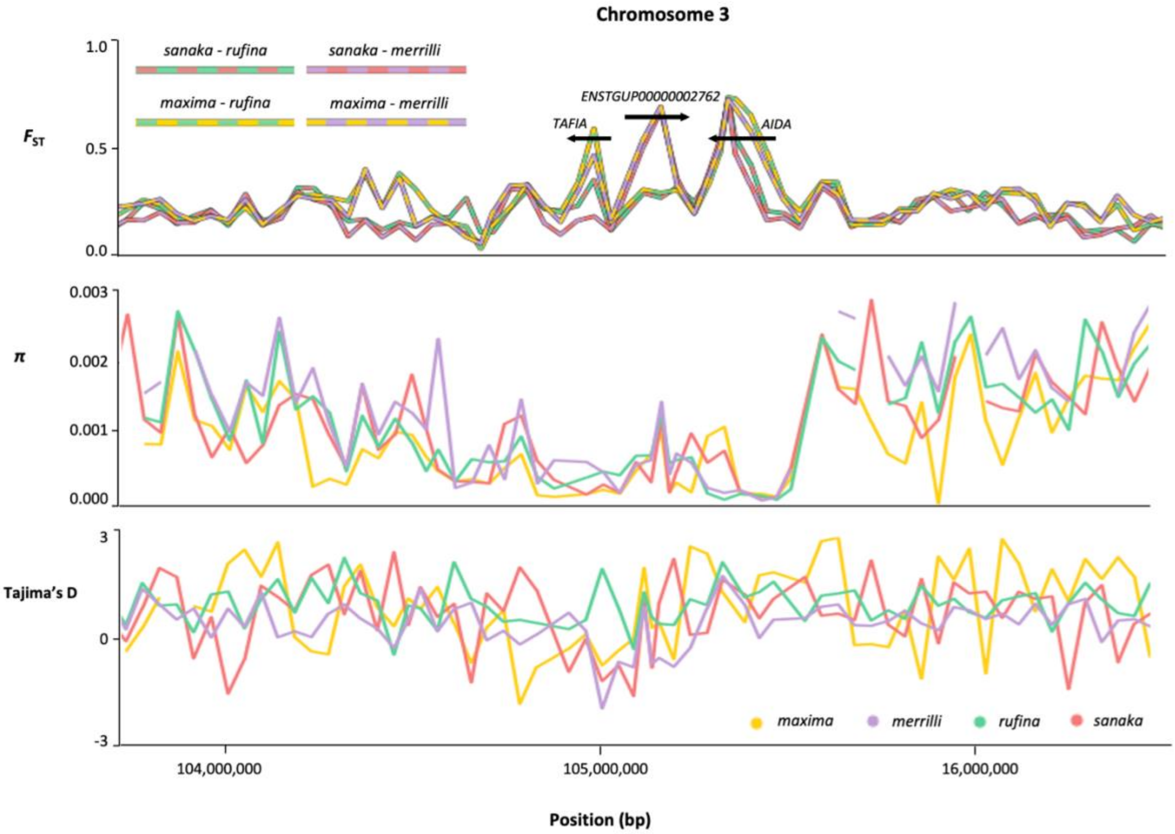

Supplementary Figure 5. Distribution of F_{ST}, π, Tajima's *D* in 25-kb windows, and genes within the divergent windows on Chromosome 3 (contig 1534).

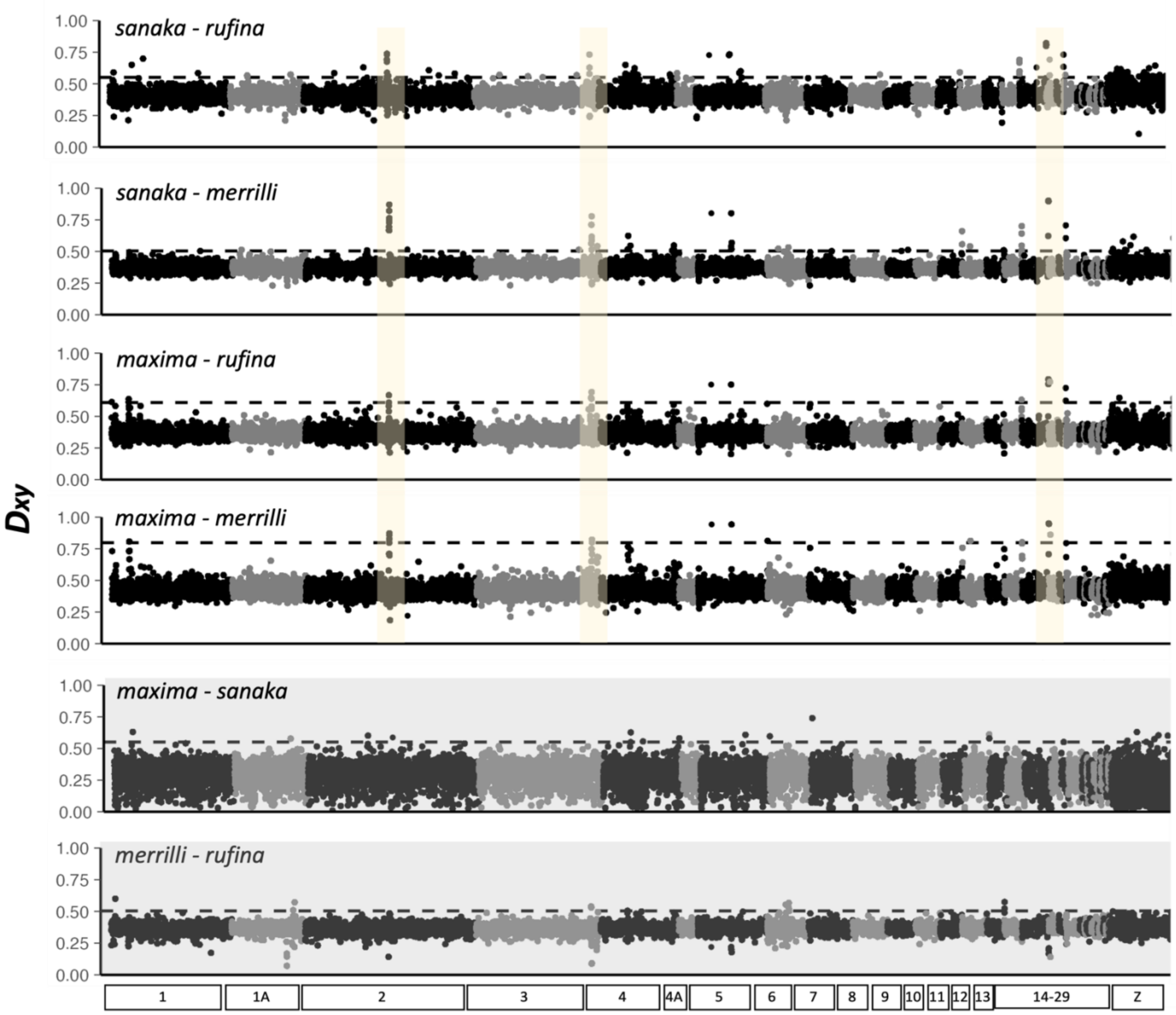

Supplementary Figure 6. Genome-wide distribution of absolute genetic divergence (*Dxy*) across 50 kb windows for pairwise comparisons between large- and smaller-bodied northern subspecies (white background) and control comparisons (grey background). The dashed line indicates the 99.9th percentile of genome-wide mean. The yellow highlighted regions indicate a subset of divergent windows on contigs 3361 (chr 2), 1534 (chr 3), and 391 (chr 17) held in common between all pairs. Chromosomes were identified by their position on the zebra finch genome.

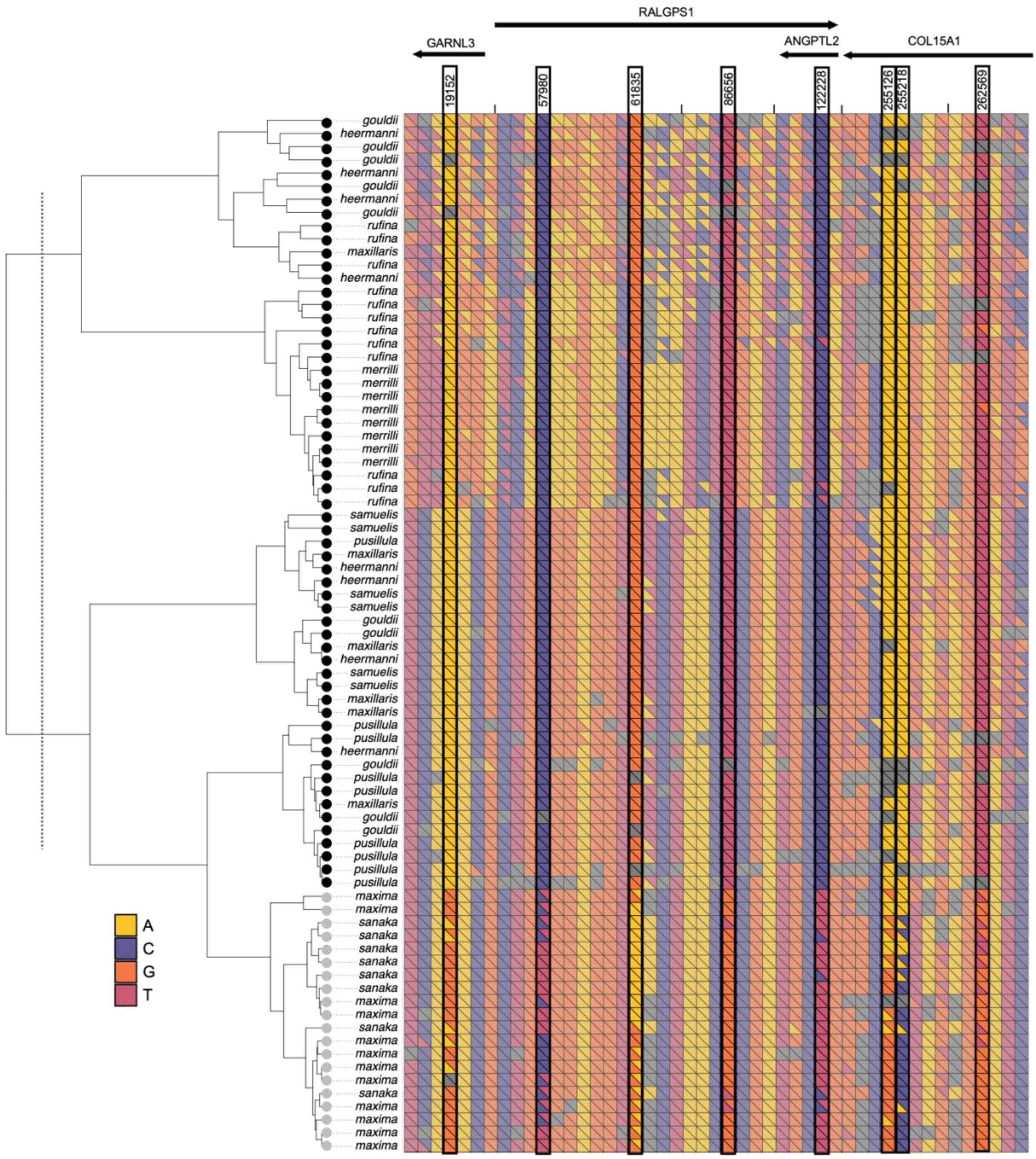

Supplementary Figure 7. Genotypes for eight highly divergent SNPs located within the peaks on contigs 391 (chr 17) and 3361 (chr 2) of *M. m. maxima* and *M. m. sanaka* (large-bodied; gray circles), and *M. m. rufina, M. m. merrilli, M. m. gouldii, M. m. heermanni, M. m. maxillaris, M. m. pusillula,* and *M. m. samuelis* (smaller- and small-bodied; black circles) for eight highly divergent SNPs located in the peaks on contigs 391 (chr 17) and 3361 (chr 2). The phylogenetic tree is reconstructed from 54 SNPs, including 1-3 SNPs up and downstream from the 8 focal SNPs, resulting in clustering by body size and geography. Positions are consecutive along contig 391 or 3361 until marked with a horizontal line to represent a break point, and genes are noted along the top. The four nucleotides are color-coded as indicated in the figure legend and missing data as grey, with the base pairs of each individual at a locus represented in the two halves of a diagonally split pixel. At the SNPs of interest, small-bodied subspecies are predominantly homozygous, while large-bodied subspecies are homozygous for the alternate allele or heterozygous.
