# Supplementary material for: Candidate genes under selection in song sparrows co-vary with climate and body mass in support of Bergmann’s Rule
Source: Nat Commun. 2023 Nov 7;14:6974. doi: 10.1038/s41467-023-42786-2 (PMC10630373; doi:10.1038/s41467-023-42786-2)
Supplement: Supplementary file 3 — Description of Additional Supplementary Files [file 41467_2023_42786_MOESM3_ESM.pdf]

Description of Additional Supplementary Files

Supplementary Data 1
Description: Table summarizing sample information for the song sparrows (*Melospiza melodia*) sequenced for this study. Table includes lab sample ID, sample ID for analysis, museum ID, subspecies designation, sampling location, collection date, mass (grams), barcode index for library prep, sequencing stats (% mapped reads, number of mapped reads, mapped paired reads, coverage, standard deviation of coverage, mapping quality, and % missing data), FIS, and PCA axes.
