# Supplementary material for: Candidate genes under selection in song sparrows co-vary with climate and body mass in support of Bergmann’s Rule
Source: Nat Commun. 2023 Nov 7;14:6974. doi: 10.1038/s41467-023-42786-2 (PMC10630373; doi:10.1038/s41467-023-42786-2)
Supplement: Supplementary file 5 — Reporting Summary [file 41467_2023_42786_MOESM5_ESM.pdf]

nature portfolio

Corresponding author(s): Katherine Carbeck

Last updated by author(s): Sep 21, 2023

Reporting Summary

Nature Portfolio wishes to improve the reproducibility of the work that we publish. This form provides structure for consistency and transparency in reporting. For further information on Nature Portfolio policies, see our Editorial Policies and the Editorial Policy Checklist.

Statistics

For all statistical analyses, confirm that the following items are present in the figure legend, table legend, main text, or Methods section.

n/a	Confirmed	
☐	☒	The exact sample size (*n*) for each experimental group/condition, given as a discrete number and unit of measurement
☐	☒	A statement on whether measurements were taken from distinct samples or whether the same sample was measured repeatedly
☐	☒	The statistical test(s) used AND whether they are one- or two-sided<br>*Only common tests should be described solely by name; describe more complex techniques in the Methods section.*
☐	☒	A description of all covariates tested
☐	☒	A description of any assumptions or corrections, such as tests of normality and adjustment for multiple comparisons
☐	☒	A full description of the statistical parameters including central tendency (e.g. means) or other basic estimates (e.g. regression coefficient) AND variation (e.g. standard deviation) or associated estimates of uncertainty (e.g. confidence intervals)
☐	☒	For null hypothesis testing, the test statistic (e.g. *F*, *t*, *r*) with confidence intervals, effect sizes, degrees of freedom and *P* value noted<br>*Give P values as exact values whenever suitable.*
☒	☐	For Bayesian analysis, information on the choice of priors and Markov chain Monte Carlo settings
☒	☐	For hierarchical and complex designs, identification of the appropriate level for tests and full reporting of outcomes
☐	☒	Estimates of effect sizes (e.g. Cohen's *d*, Pearson's *r*), indicating how they were calculated

Our web collection on statistics for biologists contains articles on many of the points above.

Software and code

Policy information about availability of computer code

Data collection	No software was used for data collection.
Data analysis	Data Filtering/Variant Discovery: FastQC v.0.11.8, AdapterRemoval V2.1.1, Bowtie2 v.2.4.2, SamTools v.1.9, Bcftools v.1.12, Picard Tools v.2.8.2, Qualimap v.2.2.1, SweeD v.3.3.2<br>Summary Statistics/population genomic analyses: R v.4.0.2, VCFtools v.0.1.14, SNPRelate v.3.17, ADMIXTURE v.1.23, Beagle v.3.3.2<br>Gene ID: R, Geneious v.11.1.5, Uniprot, Ensembl, NHGRI-EBI GWAS catalog<br>Validation: VCFtools v.0.1.14, R v.4.0.2, vegan v.2.6.4, ape v.5.7.1<br>GitHub: https://doi.org/10.5281/zenodo.8365146

For manuscripts utilizing custom algorithms or software that are central to the research but not yet described in published literature, software must be made available to editors and reviewers. We strongly encourage code deposition in a community repository (e.g. GitHub). See the Nature Portfolio guidelines for submitting code & software for further information.

Data

Policy information about availability of data

All manuscripts must include a data availability statement. This statement should provide the following information, where applicable:

- Accession codes, unique identifiers, or web links for publicly available datasets
- A description of any restrictions on data availability
- For clinical datasets or third party data, please ensure that the statement adheres to our policy

The raw sequencing data generated in this study have been deposited in the National Center for Biotechnology Information (NCBI) BioProject database under accession code PRJNA1013697 [https://www.ncbi.nlm.nih.gov/bioproject/PRJNA1013697]. Raw sequencing data for the California song sparrow subspecies used in this study are available in the NCBI BioProject database under accession code PRJNA1018990 [http://www.ncbi.nlm.nih.gov/bioproject/1018990]. Source data are also provided with this paper.

Human research participants

Policy information about studies involving human research participants and Sex and Gender in Research.

Reporting on sex and gender	N/A
Population characteristics	N/A
Recruitment	N/A
Ethics oversight	N/A

Note that full information on the approval of the study protocol must also be provided in the manuscript.

Field-specific reporting

Please select the one below that is the best fit for your research. If you are not sure, read the appropriate sections before making your selection.

☐ Life sciences ☐ Behavioural & social sciences ☒ Ecological, evolutionary & environmental sciences

For a reference copy of the document with all sections, see nature.com/documents/nr-reporting-summary-flat.pdf

Ecological, evolutionary & environmental sciences study design

All studies must disclose on these points even when the disclosure is negative.

Study description	Whole genome sequencing of 40 large- and small-bodied subspecies of song sparrows from the northern extent of their range to characterize the gneomic variation in relation to body mass, which was then compared to genotype of the five smallest subspecies endemic to California (n = 39).
Research sample	Tissue samples were obtained from the University of Alaska Museum from sites in Alaska and Northern British Columbia. Populations were chosen primarily based on availability but constitute a comprehensive variation in body size over a relatively small geographic space. Melospiza melodia maxima (n=12), M. m. sanaka (n=8), M. m. merrilli (n=8), M. m. rufina (n=12). M. m. gouldii (n = 10), M. m. heermanni (n = 8), M. m. samuelis (n = 6), M. m. pusillula (n = 9), and M. m. maxillaris (n = 6) raw sequences were obtained from Mikles et. al, 2020 as part of another study.
Sampling strategy	Tissue samples were collected from populations in close geographic proximity vary substantially in mass and temperature. California samples were used to test if genotypes of the smallest subspecies of song sparrows could be predicted by that of the small-bodied northern subspecies. No sample-size selection was performed prior to the study as samples were selected based on availability.
Data collection	Tissues and skins from the collected individuals are archived at the University of Alaska Museum. DNA was extracted using standard protocols (detailed in methods). Sequencing was done at the Cornell Institute for Biotechnology core facility and Novagene at the University of California at Davis campus.
Timing and spatial scale	All samples were collected May - September between 1997 and 2000 from Alaska to California.
Data exclusions	We filtered out variants that were not biallelic, had minor allele frequencies less than 5%, mean coverage less than 2X or more than 50X, and more than 20% missing data. This resulted in a total of 13,089,663 SNPs across the four subspecies.
Reproducibility	The raw sequencing data generated in this study have been deposited in the National Center for Biotechnology Information (NCBI) BioProject database under accession code PRJNA1013697 [https://www.ncbi.nlm.nih.gov/bioproject/PRJNA1013697]. Raw sequencing data for the California song sparrow subspecies used in this study are available in the NCBI BioProject database under accession code PRJNA1018990 [http://www.ncbi.nlm.nih.gov/bioproject/1018990]. Source data are also provided with this paper.

	Scripts and bioinformatic pipelines can be found on GitHub (https://doi.org/10.5281/zenodo.8365146) to allow for reproducibility of results. We have not re-run the pipelines due to lengthy and expensive computation time, but the bioinformatic pipelines are robust and have been used extensively.
Randomization	Samples were selected based on availability at a location. Randomization is not applicable.
Blinding	There was no experimental treatment of samples involved in this study that requires blinding. Data analysis was performed based on the analysis groups that were defined by external information (phenotype/geographic location).

Did the study involve field work? ☐ Yes ☒ No

Reporting for specific materials, systems and methods

We require information from authors about some types of materials, experimental systems and methods used in many studies. Here, indicate whether each material, system or method listed is relevant to your study. If you are not sure if a list item applies to your research, read the appropriate section before selecting a response.

Materials & experimental systems

n/a	Involved in the study
☒	☐ Antibodies
☒	☐ Eukaryotic cell lines
☒	☐ Palaeontology and archaeology
☒	☐ Animals and other organisms
☒	☐ Clinical data
☒	☐ Dual use research of concern

Methods

n/a	Involved in the study
☒	☐ ChIP-seq
☒	☐ Flow cytometry
☒	☐ MRI-based neuroimaging
